# Diagnosis of Neonatal Herpes Simplex Infection from the Placenta

**DOI:** 10.1155/2020/8898612

**Published:** 2020-11-20

**Authors:** Amanda E. Smith, Amy McKenney, Laura Rabinowitz, Anirudha Das

**Affiliations:** ^1^Department of Neonatology, Cleveland Clinic Children's Hospital, Cleveland, OH, USA; ^2^Department of Pathology, Cleveland Clinic Foundation, Cleveland, OH, USA

## Abstract

Due to a high rate of fetal demise and premature birth in intrauterine HSV infection, the outcome in neonates is usually adverse. A female preterm infant with a gestational age of 25 1/7 weeks with expected early clinical course tested positive for neonatal herpes simplex virus (HSV) 2 after the neonatologist was informed of positive immunohistochemistry for the virus on the fifth day of life by the pathologist. Pathological examination of the placenta had revealed subacute necrotizing inflammation with stromal cell necrosis suggestive of intrauterine infection, possibly ascending due to prolonged rupture of membranes. To the best of our knowledge, this is the first case wherein placental pathology indicated exposure to HSV *in utero* before the infant presented with signs or symptoms of neonatal HSV resulting in a favorable outcome for the infant. Due to the variability of presentation of intrauterine HSV infection, pathological examination of the placenta in the first 2–5 days of life in premature infants can provide clues to the diagnosis of neonatal HSV which may significantly impact the outcome.

## 1. Introduction

Neonatal herpes simplex virus (HSV) infection, defined as infection within 28 days of birth, is a rare disease that can lead to severe morbidity and mortality. The most common route of infection is intrapartum accounting for >85% of the infections, but rarely, intrauterine or postnatal transmission is also reported [1]. The rate of intrauterine infection is close to 5%, which works out to be 1 in 100,000 deliveries, a considerably rare infection [1]. *In utero* transmission can lead to fetal loss, premature delivery, or a high mortality rate of about 50% of infants despite appropriate therapy [2]. Although a triad of skin, central nervous system, and ophthalmologic presentation have been reported to be present early in the case of intrauterine infections, such a triad of clinical features were demonstrated to occur less frequently by subsequent reports [1]. In about 60–80% of mothers of infants with neonatal HSV, symptoms are absent at or before the delivery [3]. Difficulty in making an early diagnosis leads to significant morbidity and mortality in these infants. In a report by Marquez, 2/3 infants had a significant developmental delay at six months of age [1]. Therefore, making an early diagnosis can be the difference between morbidity/mortality and a favorable outcome.

We present a case where the diagnosis of intrauterine HSV infection was made initially from placental pathology and immunohistochemistry in an asymptomatic preterm infant, which prompted early antiviral therapy and a favorable outcome in the infant.

## 2. Case Presentation

The case was a female preterm infant with a gestational age of 25 1/7 weeks delivered by cesarean section (weight, 703 grams; length, 31 centimeter; and head circumference, 22 centimeters, appropriate for gestational age) for placental abruption. Membranes were ruptured for 5 days and 20 hours before delivery. The infant was admitted to the neonatal intensive care unit (NICU) and treated for respiratory distress via mechanical ventilation. The infant was initially intubated and received a dose of surfactant and extubated on day of life (DOL) 3 to continuous positive airway pressure (CPAP). The infant received antibiotics for the first 36 hours per protocol, for prolonged premature rupture of membrane (PPROM) of 6 days. Umbilical venous and arterial catheters were placed for vascular access. Maternal prenatal labs were negative, except for group B streptococcus testing, which was unknown, and the mother had no history of HSV.

**Figure 1:**
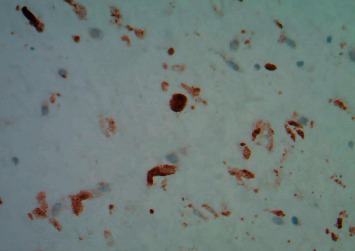
Immunohistochemical staining for HSV 1 and 2 confirms the presence of the virus (Cell Marque, rabbit polyclonal).

**Figure 2:**
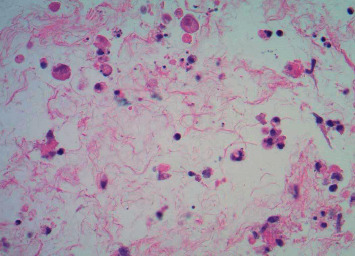
Individual cell necrosis with viral cytopathic effect seen in the subamniotic umbilical cord stroma (hematoxylin and eosin).

The placenta was sent for pathological examination due to prematurity of the infant on DOL 1. The presence of subacute necrotizing inflammation with stromal cell necrosis on the placental pathological examination suggested the possibility of HSV infection. Besides, rare plasma cells were seen in the fetal inflammatory response, which included mononuclear cells. These changes indicated the ascending pattern of HSV infection with changes primarily within the umbilical cord and chorionic plate of the placenta (Figure 1). Immunohistochemical staining for HSV 1 and 2 (Cell Marque rabbit polyclonal antibody) confirmed the presence of herpes simplex virus type 2 in the amniocytes and stromal cells of the cord and chorionic plate (Figure 2).

The pathologist notified the neonatologist regarding the positive result on DOL 5. At that time, the infant was stable on noninvasive ventilation, tolerating feeds well. Immediately, blood HSV polymerase chain reaction (PCR), HSV surface cultures, and HSV PCR of cerebrospinal fluid were sent on the baby, and the infant was started on intravenous (IV) acyclovir at 20 milligram/kilogram/dose every 12 hours. The HSV surface culture (from rectum swab) and blood HSV PCR was positive without any other symptomatic or laboratory abnormalities suggestive of any organ involvement. HSV PCR from the cerebrospinal fluid was negative. The infant was diagnosed with skin, eye, mucosa (SEM) neonatal HSV disease. The infant was treated with IV acyclovir for 14 days and then started on oral suppressive acyclovir therapy. The oral suppressive acyclovir was continued for seven and a half months since birth at a dose of 20 milligrams (mg) per kilogram (kg) twice daily. The acyclovir was then switched to valacyclovir (20 mg/kg) once daily and continued at 20 months of age. The dose was increased to twice daily when there was a skin breakout of lesions and lowered to once daily after the lesions were resolved.

**Figure 3:**
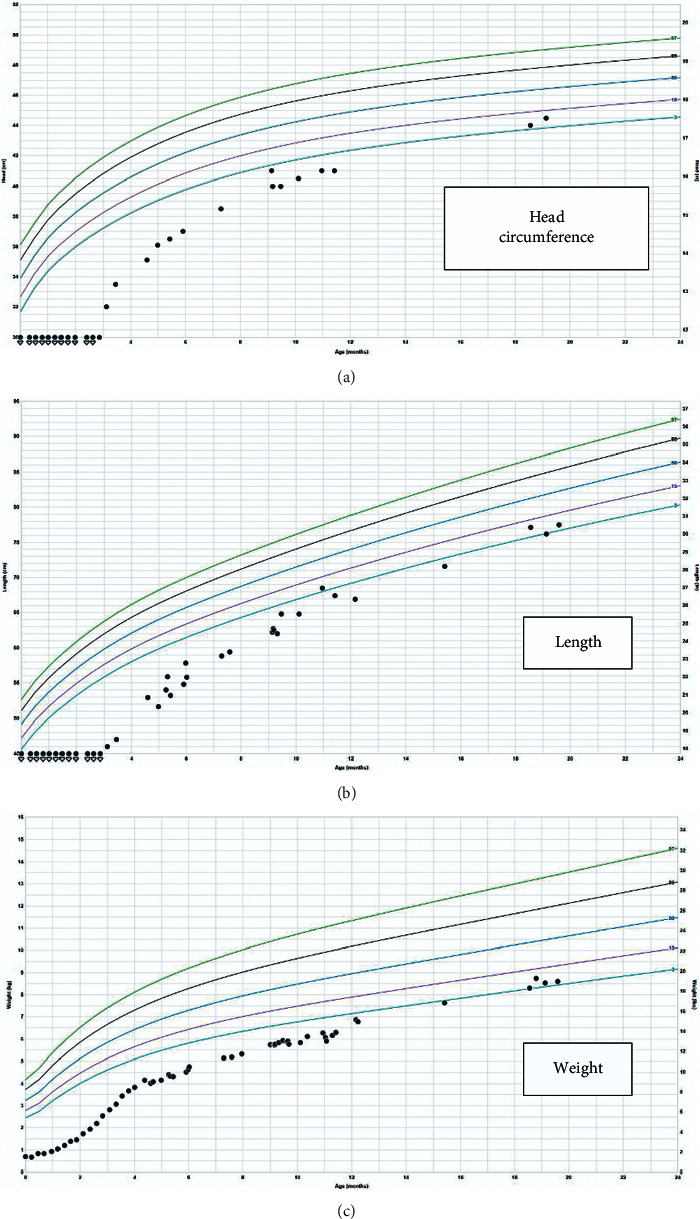
Growth in (c) weight, (b) length, and (a) head circumference of the child from birth to 20 months.

Subsequently, the infant developed unrelated renal vein thrombosis complications and renal atrophy, which were managed accordingly. The infant developed cutaneous HSV lesions whenever valacyclovir was discontinued, even for a few days. The vesicles were mostly present over the lower abdomen, back, and lower extremities. Breakout lesions occurred at least 3 times over the last 6 months. When writing this report, the child was 20 months old, on valacyclovir, and had normal developmental milestones for corrected age. The growth pattern of the child is illustrated in Figure 3.

## 3. Discussion and Review of Literature

We present a case of intrauterine HSV infection, wherein the pathologist made the diagnosis before clinical presentation in the infant, which proved to be a critical factor in rendering a favorable outcome for the infant. To the best of our knowledge, diagnosis and intervention of neonatal HSV by pathological diagnosis have not been reported previously. Due to a high rate of fetal demise and premature birth in intrauterine HSV infection, the outcome in neonates is usually adverse. This case stresses the need for careful placental examination, especially in premature infants, as clinical presentation of neonates with intrauterine HSV infection is variable.

The most common transmission of HSV at birth is through the infected birth canal, but rare intrauterine infection has also been reported. Neonates are at the highest risk of acquiring neonatal HSV infection when there is a maternal primary HSV infection in the third trimester, with an estimated 50–60% infection risk. However, two out of three women who acquire HSV during pregnancy are either asymptomatic or have nonspecific symptoms [4]. Without a maternal diagnosis or concern for HSV, neonates typically are not tested or treated for HSV infection until they become symptomatic, which is the reason for higher morbidity and mortality. In our case, if the natural progression of the disease is to be applied, the infant would most likely have presented within the first 10–14 DOL with symptoms of HSV. In this case, starting specific antiviral therapy before clinical presentation led to a favorable outcome.

About 80% of neonates with SEM disease typically present with a vesicular rash [4]. Although these infants have the best prognosis out of the three types of neonatal HSV infections, they remain at risk for recurrent skin lesions [5]. Infants with SEM only disease have low risk of developmental disability at 12 months of age [4]. Infants affected by disseminated HSV disease also present between days 10 and 12 but with respiratory and hepatic failure as well as disseminated coagulation and have the highest rate of mortality, while infants with CNS involvement typically present between days 16 and 19 with seizures, lethargy, temperature instability, poor feeding, and bulging fontanels [4, 5]. Overall, an estimated 30% of neonatal HSV infection survivors have abnormal neurological outcomes with worse outcomes in intrauterine infections. In a report of 3 cases and a review of 64 cases of intrauterine infection, Marquez notes mortality in 29/64 (45%), including 4 stillbirths and developmental delay in 13/64 (20%) infants [1].

Early treatment with high-dose acyclovir decreases the risk of progression to disseminated disease and/or CNS involvement and can also reduce the disease's severity and improve long-term outcomes [4, 5]. In this case, the infant was asymptomatic since birth. A suspicion and a positive placental immunohistochemical identification of the virus prompted the pathologist to contact the neonatologist, leading to further confirmatory testing and starting of appropriate therapy. The timely alerting of the neonatology team by the pathologist, in this case, resulted in a favorable outcome.

The identification of HSV in the placenta is uncommon. This suggests *in utero* infection as a primary mechanism of transmission rather than infection from the birth canal. In many neonatal HSV infection cases, the placenta is never examined as the infants appear well initially. Transplacental infection via the maternal bloodstream is often cited as the primary mechanism of *in utero* HSV transmission, common to other viruses such as cytomegalovirus. A few case reports have recently described intrauterine HSV infection with devastating consequences involving the brain and the heart [6, 7]. However, HSV can have an ascending pattern of infection in the placenta, similar to ascending bacterial infections with chorioamnionitis [2]. Pathological examination of the placenta in intrauterine HSV infection can reveal villous necrosis, necrotizing chorioamnionitis with or without infiltration of the blood vessels [2]. In a study by Finger-Jardim, the HSV-2 detection rate in placental samples was found to be 9.0% (*n* = 18), all of whom were asymptomatic did not report genital herpes. They demonstrated that delay of delivery of ≥6 hours from rupture of membrane had an approximately fourfold risk of HSV-2 infection in the placental tissue [8]. Our case had subacute necrotizing inflammation with stromal cell necrosis and infiltration of the placenta, which led to the suspicion of intrauterine HSV and positive immunohistochemical staining. This very likely resulted from an ascending transcervical infection also contributed by the premature prolonged rupture of membranes.

## 4. Conclusion

To the best of our knowledge, this is the first case wherein placental pathology indicated exposure to HSV *in utero* before the infant presented with signs or symptoms of neonatal HSV, resulting in a favorable outcome for the infant. Due to the variability of presentation of intrauterine HSV infection, pathological examination of the placenta in the first 2–5 days of life in premature infants can provide clues to the diagnosis of neonatal HSV, which may significantly impact the outcome. Further research is needed to elucidate the importance of early pathological examination guiding testing and antiviral treatment to decrease morbidity, mortality, and neurologic impairment of neonates affected by HSV infection.
